# Supplementary material for: Homophily modulates double descent generalization in graph convolution networks
Source: Proc Natl Acad Sci U S A. 2024 Feb 12;121(8):e2309504121. doi: 10.1073/pnas.2309504121 (PMC10895367; doi:10.1073/pnas.2309504121)
Supplement: Supplementary file 1 — Appendix 01 (PDF) [file pnas.2309504121.sapp.pdf]

Supporting Information for

Homophily modulates double descent generalization in graph convolution networks

Cheng Shi, Liming Pan, Hong Hu and Ivan Dokmanić

Liming Pan.
E-mail: panlm99@gmail.com,
Ivan Dokmanić.
E-mail: ivan.dokmanic@unibas.ch

This PDF file includes:

Supporting text
Figs. S1 to S3
Table S1
SI References

Supporting Information Text

1. Sketch of the derivation

A. Replica method. We now outline the replica-based derivations. We first consider one-layer GCN $\boldsymbol{P}(\boldsymbol{A}) = \boldsymbol{A}$ to show the main idea. The training and test risks in this case is given by Eq. (5) in the main text. We further extend the analysis when self-loops are included in Appendix 2.

We begin by defining the *augmented partition function*,

$$Z_\beta(\boldsymbol{A}, \boldsymbol{X}) = \int \mathrm{d}\boldsymbol{w} \exp\left(-\beta H(\boldsymbol{w}) + t_0 \beta O_{\text{train}}(\boldsymbol{w}) + t_1 \beta O_{\text{test}}(\boldsymbol{w})\right),$$

where β is the inverse temperature. The Hamiltonian in the above equation reads

$$H(\boldsymbol{w}) = \|\boldsymbol{I}_{\text{train}} \boldsymbol{A}\boldsymbol{X}\boldsymbol{w} - \boldsymbol{I}_{\text{train}}\boldsymbol{y}\|_2^2 + \tau r \|\boldsymbol{w}\|_2^2, \qquad \text{[S.1]}$$

which is the loss Eq. (4) scaled by $N\tau$. The "observables" O_{train} and O_{test} (the scaled training and test risks) are the quantities we are interested in:

$$O_{\text{train}}(\boldsymbol{w}) = \|\boldsymbol{I}_{\text{train}} \boldsymbol{A}\boldsymbol{X}\boldsymbol{w} - \boldsymbol{I}_{\text{train}}\boldsymbol{y}\|_2^2, \quad O_{\text{test}}(\boldsymbol{w}) = \|\boldsymbol{I}_{\text{test}} \boldsymbol{A}\boldsymbol{X}\boldsymbol{w} - \boldsymbol{I}_{\text{test}}\boldsymbol{y}\|_2^2.$$

When the inverse temperature β is small, the Gibbs measure $Z_\beta^{-1}(A, X)\exp(-\beta H(\boldsymbol{w}))\,\mathrm{d}\boldsymbol{w}$ is diffuse; when $\beta \to \infty$, the Gibbs measure converges to an atomic measure concentrated on the unique solution of Eq. (4). That is to say, for $t_0 = t_1 = 0$, we can write

$$\boldsymbol{w}^* = \lim_{\beta\to\infty} \int \boldsymbol{w}\, \mathbb{P}_\beta(\boldsymbol{w}; \boldsymbol{A}, \boldsymbol{X})\mathrm{d}\boldsymbol{w}, \quad \text{where} \quad \mathbb{P}_\beta(\boldsymbol{w}; \boldsymbol{A}, \boldsymbol{X}) = \frac{1}{Z_\beta(\boldsymbol{A}, \boldsymbol{X})} \exp\left(-\beta H\left(\boldsymbol{w}\right)\right).$$

The idea is to compute the values of the observables in the large system limit at a finite temperature and then take the limit $\beta \to \infty$. To this end, we define the *free energy density* f_β corresponding to the augmented partition function,

$$f_\beta := -\lim_{N\to\infty} \frac{1}{N\beta} \mathbb{E}_{\boldsymbol{A},\boldsymbol{X}} \ln Z_\beta(\boldsymbol{A}, \boldsymbol{X}). \qquad \text{[S.2]}$$

The expected risks can be computed as

$$R_{\text{train}} = -\frac{1}{\tau} \lim_{\beta\to\infty} \frac{\partial f_\beta}{\partial t_0}\bigg|_{t_0=0, t_1=0}, \quad R_{\text{test}} = -\frac{1}{1-\tau} \lim_{\beta\to\infty} \frac{\partial f_\beta}{\partial t_1}\bigg|_{t_0=0, t_1=0}. \qquad \text{[S.3]}$$

Although $\ln Z_\beta(\boldsymbol{A}, \boldsymbol{B})/N$ concentrates for large N, a direct computation of the quenched average Eq. (S.2) is intractable. We now use the *replica trick* which takes the expectation inside logarithm, or replaces the quenched average by annealed average in physics jargon:

$$\mathbb{E}_{\boldsymbol{A},\boldsymbol{X}} \ln Z_\beta\left(\boldsymbol{A}, \boldsymbol{X}\right) = \lim_{n\to 0} \frac{\ln \mathbb{E}_{\boldsymbol{A},\boldsymbol{X}} Z_\beta^n\left(\boldsymbol{A}, \boldsymbol{X}\right)}{n}. \qquad \text{[S.4]}$$

The main idea of the replica method is to first compute $\mathbb{E}_{\boldsymbol{A},\boldsymbol{X}} Z_\beta^n$ for integer n by interpreting $Z_\beta^n = (Z_\beta)_1 (Z_\beta)_2 \dots (Z_\beta)_n$ as the product of n partition functions for n independent configurations $\{\boldsymbol{w}^a\}_{a=1}^n$. Then we obtain $\mathbb{E}_{\boldsymbol{A},\boldsymbol{X}} \ln Z_\beta$ by taking the limit $n \to 0$ in Eq. (S.4), even though the formula for $\ln \mathbb{E}_{\boldsymbol{A},\boldsymbol{X}} {Z_\beta}^n$ is valid only for integer n. The expectation of replicated partition function reads

$$\begin{aligned}\mathbb{E}_{\boldsymbol{A},\boldsymbol{X}} Z_\beta^n(\boldsymbol{A}, \boldsymbol{X}) &= \mathbb{E}_{\boldsymbol{A},\boldsymbol{X}} \left(Z_\beta(\boldsymbol{A}, \boldsymbol{X})\right)_1 \times \dots \times \left(Z_\beta(\boldsymbol{A}, \boldsymbol{X})\right)_n \\ &= \int \prod_{a=1}^n \mathrm{d}\boldsymbol{w}^a \mathbb{E}_{\boldsymbol{A},\boldsymbol{X}} \exp\left(\sum_{a=1}^n \left(-\|\boldsymbol{I}_\beta \boldsymbol{A}\boldsymbol{X}\boldsymbol{w}^a - \boldsymbol{I}_\beta \boldsymbol{y}\|_2^2\right)\right) \exp(-\beta\tau r \|\boldsymbol{w}^a\|_2^2),\end{aligned} \qquad \text{[S.5]}$$

where we denote $\boldsymbol{I}_\beta \overset{\text{def}}{=} \sqrt{\beta - \beta t_0} \boldsymbol{I}_{\text{train}} + \sqrt{-\beta t_1} \boldsymbol{I}_{\text{test}}$. We first keep $\boldsymbol{X}$ fixed and take the expectation over $\boldsymbol{A}$. Directly computing the expectation over the binary graph matrix $\boldsymbol{A} \sim \mathcal{A}^{\text{bs}}$ is non-trivial. To make progress, we average over $\boldsymbol{A} \sim \mathcal{A}^{\text{gn}}$ (instead cf. Eq. (15)). In Appendix B we show that this Gaussian substitution does not change the free energy density, ultimately yielding the same risks and accuracies as detailed in Conjecture 1.

Letting $\boldsymbol{\sigma}^a = \boldsymbol{X}\boldsymbol{w}^a$, the elements of $\boldsymbol{A}^{\text{gn}}\boldsymbol{\sigma}^a$ are now jointly Gaussian for any fixed $\boldsymbol{\sigma}^a$ and $C = \frac{1}{nN} \ln \mathbb{E}_{\boldsymbol{A}} \exp\left(\sum_{a=1}^n \left(-\|\boldsymbol{I}_\beta \boldsymbol{A}\boldsymbol{\sigma}^a - \boldsymbol{I}_\beta \boldsymbol{y}\|^2\right)\right)$ can be computed by multivariate Gaussian integration. It is not hard to see that C depends only on a vector $\boldsymbol{m} \in \mathbb{R}^n$ and a matrix $\boldsymbol{Q} \in \mathbb{R}^{n\times n}$ defined as

$$\boldsymbol{m}_a = \boldsymbol{y}^T \boldsymbol{\sigma}^a / N, \quad \text{and} \quad \boldsymbol{Q}_{ab} = (\boldsymbol{\sigma}^a)^\intercal \boldsymbol{\sigma}^b / N. \qquad \text{[S.6]}$$

In statistical physics these quantities are called the *order parameters*. We then define

$$\begin{aligned}C(\boldsymbol{m}, \boldsymbol{Q}) &= \frac{1}{nN} \ln \mathbb{E}_{\boldsymbol{A}}\, c(\boldsymbol{A}) \\ &= \frac{1}{nN} \ln \mathbb{E}_{\boldsymbol{A}} \exp\left(-\sum_a \|\boldsymbol{I}_\beta \boldsymbol{A}\boldsymbol{\sigma}^a - \boldsymbol{I}_\beta \boldsymbol{y}\|_2^2\right).\end{aligned} \qquad \text{[S.7]}$$

Using the Fourier representation of the Dirac delta function $\delta(t-t_0)=\frac{1}{2\pi}\int \mathrm{d}\omega \exp(i\omega(t-t_0))$, we have

$$
\begin{aligned}
\delta\left(N\boldsymbol{Q}_{ab}-(\boldsymbol{w}^a)^\intercal \boldsymbol{X}^\intercal \boldsymbol{X}\boldsymbol{w}^b\right) &= \frac{1}{2\pi}\int d\widehat{\boldsymbol{Q}}_{ab}\exp\left(i\widehat{\boldsymbol{Q}}_{ab}\left(N\boldsymbol{Q}_{ab}-(\boldsymbol{w}^a)^\intercal \boldsymbol{X}^\intercal \boldsymbol{X}\boldsymbol{w}^b\right)\right),\\
\delta\left(N\boldsymbol{m}_a-\boldsymbol{y}^\intercal \boldsymbol{X}\boldsymbol{w}^a\right) &= \frac{1}{2\pi}\int d\widehat{\boldsymbol{m}}_a\exp\left(i\widehat{\boldsymbol{m}}_a\left(N\boldsymbol{m}_a-\boldsymbol{y}^\intercal \boldsymbol{X}\boldsymbol{w}^a\right)\right),
\end{aligned}
\tag{S.8}
$$

so that Eq. (S.5) becomes

$$
\begin{aligned}
\mathbb{E}_{\boldsymbol{A},\boldsymbol{X}} Z_\beta^n(\boldsymbol{A},\boldsymbol{X}) = \left(\frac{iN}{2\pi}\right)^{\frac{n^2+3n}{2}} &\int \prod_{a\le b}\mathrm{d}\boldsymbol{Q}_{ab}\prod_{a\le b}\mathrm{d}\widehat{\boldsymbol{Q}}_{ab}\prod_a \mathrm{d}\boldsymbol{m}_a\prod_a \mathrm{d}\widehat{\boldsymbol{m}}_a\\
&\times\exp\left(nNC\left(\boldsymbol{m},\boldsymbol{Q}\right)\right)\times\exp\left(nNE\left(\widehat{\boldsymbol{m}},\widehat{\boldsymbol{Q}}\right)\right)\\
&\times\exp\left(N\sum_{a\le b}\widehat{\boldsymbol{Q}}_{ab}\boldsymbol{Q}_{ab}+N\sum_a\widehat{\boldsymbol{m}}_a\boldsymbol{m}_a\right),
\end{aligned}
\tag{S.9}
$$

where

$$
\begin{aligned}
E(\widehat{\boldsymbol{m}},\widehat{\boldsymbol{Q}}) &= \frac{1}{nN}\ln\mathbb{E}_{\boldsymbol{X}}\ e(\boldsymbol{X})\\
&= \frac{1}{nN}\ln\mathbb{E}_{\boldsymbol{X}}\int\prod_{a=1}^n \mathrm{d}\boldsymbol{w}_a\exp\left(-\sum_{a\le b}\widehat{\boldsymbol{Q}}_{ab}\left(\boldsymbol{w}^a\right)^T\boldsymbol{X}^\intercal\boldsymbol{X}\boldsymbol{w}^b-\sum_a\widehat{\boldsymbol{m}}_a\boldsymbol{y}^\intercal\boldsymbol{X}\boldsymbol{w}^a-\tau r\beta\sum_a\|\boldsymbol{w}_a\|_2^2\right).
\end{aligned}
\tag{S.10}
$$

In Eq. (S.9) and Eq. (S.10), we apply the change of variables $i\boldsymbol{Q}_{ab}\to\boldsymbol{Q}_{ab}$, $i\boldsymbol{m}_a\to\boldsymbol{m}_a$. Note that while Eq. (S.7) still depends on $\boldsymbol{X}$, we do not average over it. As $\boldsymbol{A}$ and $\boldsymbol{X}$ appear as a product in Eq. (S.4), it is not straightforward to average over them simultaneously. We average over $\boldsymbol{A}$ first to arrive at Eq. (S.9), and then find that Eq. (S.7)-Eq. (S.10) are self-averaging, meaning that they concentrate around their expectation as $N\to\infty$. Ultimately, this allows us to isolate the randomness in $\boldsymbol{A}$ and $\boldsymbol{X}$. It also allows the possibility to adapt our framework to other graph filters by simply replacing $\boldsymbol{A}$ by $\boldsymbol{P}(\boldsymbol{A})$ in Eq. (S.7). Since Eq. (4) has a unique solution, we take the replica symmetry assumption where the order parameters are structured as

$$
\boldsymbol{m}=m\mathbf{1}_n,\quad \widehat{\boldsymbol{m}}=\widehat{m}\mathbf{1}_n,\quad \boldsymbol{Q}=q\boldsymbol{I}_n+p\mathbf{1}_n\mathbf{1}_n^\intercal,\qquad \widehat{\boldsymbol{Q}}=\widehat{q}\boldsymbol{I}_n+\widehat{p}\mathbf{1}_n\mathbf{1}_n^\intercal. \tag{S.11}
$$

In the limit $N\to\infty$, $n\to0$, we are only interested in the leading order contributions to $C(\boldsymbol{m},\boldsymbol{Q})$ so we write (with a small abuse of notation),

$$
\begin{aligned}
C(m,p,q) =&\frac{\tau\left(2\beta(1-t_0)q+\lambda m\right)^2}{2q\left(2\beta(1-t_0)q+1\right)}-\beta(1-t_0)\tau-\frac{\tau}{2}\frac{2\beta(1-t_0)p}{2\beta(1-t_0)q+1}-\frac{\tau\lambda^2m^2}{2q}\\
&+\frac{(1-\tau)\left(-2t_1q+\lambda m\right)^2}{2q\left(-2t_1+1\right)}+t_1\left(1-\tau\right)-\frac{(1-\tau)}{2}\frac{-2t_1p}{-2t_1q+1}\\
&-\frac{(1-\tau)\,\lambda^2m^2}{2q}+o(1)+\beta o_\beta(1).^{*}
\end{aligned}
\tag{S.12}
$$

Similarly, we have

$$
E(\widehat{m},\widehat{p},\widehat{q})=\frac{1}{2(2\widehat{q}+\widehat{p})\gamma}\left(\widehat{p}\,(1-\widehat{r}T)+\gamma\widehat{m}^2\left(1-\frac{T\widehat{r}+\gamma-1}{T^2\mu\widehat{r}+(\gamma-1)T\mu+\gamma}\right)\right)+o(1)+\beta o_\beta(1), \tag{S.13}
$$

where

$$
T=\frac{1}{2\widehat{r}}\left(1-\widehat{r}-\gamma+\sqrt{1+\widehat{r}^2+2\widehat{r}+2\gamma\widehat{r}-2\gamma+\gamma^2}\right)\quad\text{and}\quad \widehat{r}=\frac{2\tau r}{2\widehat{q}+\widehat{p}}. \tag{S.14}
$$

We give the details of the derivation of C in Appendix B and of E in Appendix C.

When $r\to0$, we have $T\to\frac{1}{1-\gamma}$, and

$$
E(\widehat{m},\widehat{p},\widehat{q})=\frac{1}{2}\left(\frac{\widehat{m}^2}{2\widehat{q}+\widehat{p}}\right)\left(\frac{\mu+1}{\gamma+\mu}\right)-\frac{1}{2\gamma}\frac{\widehat{p}}{(2\widehat{q}+\widehat{p})}+o(1). \tag{S.15}
$$

We can now compute Eq. (S.9) by integrating only on 6 parameters: m, $\widehat{m}$, q, $\widehat{q}$, p, and $\widehat{p}$. For $N\to\infty$, the integral can be computed via the saddle point method:

$$
f_\beta=\frac{1}{\beta}\operatorname*{extr}_{\substack{m,\widehat{m},\\ q,\widehat{q},p,\widehat{p}}}\lim_{n\to0}\lim_{N\to\infty}C(m,p,q)+E(\widehat{m},\widehat{p},\widehat{q})+(q+p)\left(\widehat{q}+\widehat{p}\right)-\frac{1}{2}p\widehat{p}+m\widehat{m}. \tag{S.16}
$$

*We use the asymptotic notation $o(1)$ for deterministic and random quantities which vanish in the limit $n\to0$, $N\to\infty$ in a suitable sense. We similarly use $o_\beta(1)$ in the limit $\beta\to\infty$. The order parameters m,p,q in Eq. (S.12) as well as $\hat{m},\hat{p},\hat{q}$ in Eq. (S.15) scale in linear order of β or a polynomial of β.

The stationary point satisfies

$$\frac{\partial f_\beta}{\partial m} = \frac{\partial f_\beta}{\partial \widehat{m}} = \frac{\partial f_\beta}{\partial p} = \frac{\partial f_\beta}{\partial \widehat{p}} = \frac{\partial f_\beta}{\partial q} = \frac{\partial f_\beta}{\partial \widehat{q}} = 0. \tag{S.17}$$

When $\beta \to \infty$, the stationary point exists only if $m, \widehat{m}, p, \widehat{p}, q, \widehat{q}$ scale as

$$p = \mathcal{O}(1), \ q = \mathcal{O}\left(\frac{1}{\beta}\right), \ \widehat{p} = \mathcal{O}\left(\beta^2\right), \ 2\widehat{q} + \widehat{p} = \mathcal{O}(\beta), \ m = \mathcal{O}(1), \ \widehat{m} = \mathcal{O}(\beta).$$

We thus reparameterize them as

$$p \to p, \ \beta q \to q, \ \frac{\widehat{p}}{\beta^2} \to \widehat{p}, \ \frac{1}{\beta}(2\widehat{q} + \widehat{p}) \to \widehat{q}, \ m \to m, \ \frac{1}{\beta}\widehat{m} \to \widehat{m}.$$

Ignoring the small terms which vanish when $n \to 0, N \to \infty, \beta \to \infty$, and denoting $f = \lim_{\beta \to \infty} f_\beta$, we get

$$\begin{aligned} f = \operatorname*{extr}_{\substack{m, \widehat{m}, \\ q, \widehat{q}, p, \widehat{p}}} \quad & g(t_0 - 1, \tau) + g(t_1, 1 - \tau) + \frac{1}{2}\left(\widehat{q}p + q\widehat{p} + 2m\widehat{m}\right) \\ & + \frac{1}{2}\frac{\widehat{m}^2}{\widehat{q}}\left(1 - \frac{T\widehat{r} + \gamma - 1}{T^2\mu\widehat{r} + (\gamma - 1)T\mu + \gamma}\right) - \frac{1}{2\gamma}\frac{\widehat{p}(1 - \widehat{r}T)}{\widehat{q}}, \end{aligned} \tag{S.18}$$

where

$$g(t, \tau) = t\tau - \frac{\tau t p}{2tq + 1} - \frac{\tau\lambda^2 m^2}{2q} + \frac{\tau\left(2tq + \lambda m\right)^2}{2q\left(2tq + 1\right)},$$

and $\widehat{r} = \frac{2\tau r}{\widehat{q}}$. We denote by m^*, p^*, q^*, $\widehat{m}^*$, $\widehat{p}^*$, $\widehat{q}^*$ the stationary point in Eq. (S.18) and substitute into Eq. (S.3) yields the risks in 17.

We analyze the connection between the stationary point and $\boldsymbol{w}^*$. As $\boldsymbol{w}^*$ is the unique solution in Eq. (4), and from the definition of order parameters Eq. (S.6), stationarity in Eq. (S.18) implies that

$$\begin{aligned} p^* &= \lim_{N \to \infty} \mathbb{E}_{\boldsymbol{A}, \boldsymbol{X}}\left[\boldsymbol{w}^{*T}\boldsymbol{X}^T\boldsymbol{X}\boldsymbol{w}^*/N\right], \\ m^* &= \lim_{N \to \infty} \mathbb{E}_{\boldsymbol{A}, \boldsymbol{X}}\left[\boldsymbol{y}^T\boldsymbol{X}\boldsymbol{w}^*/N\right]. \end{aligned} \tag{S.19}$$

Let $\boldsymbol{A}_{\text{train}} \in \mathbb{R}^{F \times N}$ be the selection of rows from $\boldsymbol{A}$ corresponding to i-th row for all $i \in V_{\text{train}}$ and $\boldsymbol{A}_{\text{test}} \in \mathbb{R}^{(N-F) \times N}$ be the selection of rows corresponding to i-th row for all $i \in V_{\text{test}}$. The neural network output for the test nodes reads $\boldsymbol{h}_{\text{test}} = \boldsymbol{A}_{\text{test}}\boldsymbol{\sigma}^*$, where $\boldsymbol{\sigma}^* = \boldsymbol{X}\boldsymbol{w}^*$. Since we work with a non-symmetric Gaussian random matrix $\boldsymbol{A} \sim \mathcal{A}^{\text{gn}}$ as our graph matrix, $\boldsymbol{A}_{\text{test}}$ is independent of $\boldsymbol{A}_{\text{train}}$ and $\boldsymbol{\sigma}^*$ (Note $\boldsymbol{\sigma}^* = X\boldsymbol{w}^*$ depends on $\boldsymbol{A}_{\text{train}}$). Therefore, for any fixed $\boldsymbol{A}_{\text{train}}$ and $\boldsymbol{X}$ but random $\boldsymbol{A}_{\text{test}}$, the network outputs for test nodes are jointly Gaussian,

$$\boldsymbol{A}_{\text{test}}\boldsymbol{\sigma}^* \sim \mathcal{N}\left(\frac{\lambda\boldsymbol{y}^\intercal\boldsymbol{\sigma}^*}{N}\boldsymbol{y}, (\boldsymbol{\sigma}^*)^\intercal\boldsymbol{\sigma}^*\boldsymbol{I}_N\right).$$

Combining this with the results from Eq. (S.19), we obtain the test accuracy as

$$\text{ACC} = \mathbb{P}(x > 0), \quad \text{where} \quad x \sim \mathcal{N}(\lambda m^*, p^*) > 0. \tag{S.20}$$

B. Computation of $C(\cdot)$. Recall that in Eq. (S.7) we define

$$G(\mathcal{A}) := \frac{1}{nN}\ln \mathbb{E}_{\boldsymbol{A} \sim \mathcal{A}}\, c(\boldsymbol{A}). \tag{S.21}$$

In this section, we begin by computing $G(\mathcal{A}^{\text{gn}}) = C(m, p, q)$ in Eq. (S.12) where $\mathcal{A}^{\text{gn}}$ denotes the distribution of non-symmetric Gaussian spiked matrices Eq. (15). We then show that the symmetry does not influence the value of Eq. (S.21), i.e., $G(\mathcal{A}^{\text{gn}})/\beta = G(\mathcal{A}^{\text{gs}})/\beta$ when $N, d, \beta \to \infty$ and $d/N, n \to 0$. Finally, we show that the Gaussian substitution for the binary adjacency matrix does not influence the corresponding free energy density, which ultimately leads to the same risks and accuracies under different adjacency matrices (Conjecture 1).

Let's first concatenate $\{\boldsymbol{\sigma}^a\}_{a=1}^n$ as

$$\tilde{\boldsymbol{\sigma}} = \left[(\boldsymbol{\sigma}^1)_1, \cdots, (\boldsymbol{\sigma}^a)_1, \cdots, (\boldsymbol{\sigma}^n)_1, (\boldsymbol{\sigma}^1)_2, \cdots, (\boldsymbol{\sigma}^a)_2, \cdots, (\boldsymbol{\sigma}^n)_2, \cdots, (\boldsymbol{\sigma}^n)_N\right]^\intercal.$$

Then we can rewrite Eq. (S.21) in vector form

$$G(\mathcal{A}) = \frac{1}{nN}\ln \mathbb{E}_{\boldsymbol{A}} \exp\left(-\|((\boldsymbol{I}_\beta\boldsymbol{A}) \otimes \boldsymbol{1}_n)\,\tilde{\boldsymbol{\sigma}} - (\boldsymbol{I}_\beta y) \otimes \boldsymbol{1}_n\|_2^2\right), \tag{S.22}$$

where $\otimes$ is the Kronecker product. By the central limit theorem, when $N \to \infty$, the vectors $(\boldsymbol{A} \otimes \boldsymbol{1}_n)\tilde{\boldsymbol{\sigma}}$ for $\boldsymbol{A} \sim \mathcal{A}^{\mathrm{gn}}$, $\boldsymbol{A} \sim \mathcal{A}^{\mathrm{bn}}$, $\boldsymbol{A} \sim \mathcal{A}^{\mathrm{gs}}$ and $\boldsymbol{A} \sim \mathcal{A}^{\mathrm{bs}}$ all converge in distribution to Gaussian random vectors. Letting $\boldsymbol{\mu}(\mathcal{A})$ and $\boldsymbol{\Sigma}(\mathcal{A})$ be the mean and the covariance of $\boldsymbol{A} \sim \mathcal{A}$, we get

$$
\begin{aligned}
G(\mathcal{A}) =& \frac{1}{nN}\Bigg(\ln \frac{1}{\sqrt{\det\left(\boldsymbol{I}_{nN} + 2\boldsymbol{I}_{n\beta}^2 \boldsymbol{\Sigma}(\mathcal{A})\right)}} - \frac{1}{2}\boldsymbol{\mu}^{\intercal}(\mathcal{A})\,\boldsymbol{\Sigma}^{-1}(\mathcal{A})\,\mu(\mathcal{A}) - (y \otimes \boldsymbol{1}_n)^{\intercal} \boldsymbol{I}_{n\beta}^2 (\boldsymbol{y} \otimes \boldsymbol{1}_n) \\
&+ \left(\frac{1}{2}\left(2(\boldsymbol{y} \otimes \boldsymbol{1}_n)^{\intercal} \boldsymbol{I}_{n\beta}^2 + \boldsymbol{\mu}^{\intercal}(\mathcal{A})\,\boldsymbol{\Sigma}^{-1}(\mathcal{A})\right)\left(\boldsymbol{\Sigma}^{-1}(\mathcal{A}) + 2\boldsymbol{I}_{n\beta}^2\right)^{-1} \right. \\
&\left. \times \left(2\boldsymbol{I}_{n\beta}^2 \boldsymbol{y} \otimes \boldsymbol{1}_n + \boldsymbol{\Sigma}^{-1}(\mathcal{A})\,\boldsymbol{\mu}(\mathcal{A})\right)\right)\Bigg) + o(1)
\end{aligned}
\qquad [\text{S.23}]
$$

where $\boldsymbol{I}_{n\beta} = \boldsymbol{I}_{\beta} \otimes \boldsymbol{I}_n$. The vanishing lower-order term $o(1)$ comes from the tails in the central limit theorem and it is thus absent when $\mathcal{A} = \mathcal{A}^{\mathrm{gn}}$. In this case we have

$$
\boldsymbol{\mu}(\mathcal{A}^{\mathrm{gn}}) = \lambda \boldsymbol{y} \otimes \boldsymbol{m}, \quad \boldsymbol{\Sigma}(\mathcal{A}^{\mathrm{gn}}) = \boldsymbol{I}_N \otimes \boldsymbol{Q}, \qquad [\text{S.24}]
$$

with $\boldsymbol{m}$ and $\boldsymbol{Q}$ defined in Eq. (S.6). Leveraging the replica symmetric assumption Eq. (S.11), we compute the determinant term in Eq. (S.23) as

$$
\begin{aligned}
\frac{1}{nN}\det\left(\boldsymbol{I}_{nN} + 2\boldsymbol{I}_{n\beta}^2 \boldsymbol{\Sigma}(\mathcal{A}^{\mathrm{gn}})\right) &= \tau \ln\left(2\beta(1-t_0)q + 1\right) + \tau \frac{2\beta(1-t_0)np}{2\beta(1-t_0)q+1} \\
&\quad + (1-\tau)\ln\left(2(-\beta t_1)q + 1\right) + (1-\tau)\frac{2(-\beta t_1)p}{2(-\beta t_1)q+1} \\
&= \tau \frac{2\beta(1-t_0)p}{2\beta(1-t_0)q+1} + (1-\tau)\frac{2(-\beta t_1)p}{2(-\beta t_1)q+1} + o(1) \\
&\quad + \tau \ln\left(2\beta(1-t_0)q+1\right) + (1-\tau)\ln\left(2(-\beta t_1)q+1\right).
\end{aligned}
\qquad [\text{S.25}]
$$

The $o(1)$ in the third line comes from the approximation $\frac{1}{n}\ln(1+n) = 1 + o(1)$. The last two terms in Eq. (S.25) do not increase with β and can thus be neglected in the limit $\beta \to \infty$ when computing $G(\mathcal{A})/\beta$: they give rise to $\beta o_{\beta}(1)$ in Eq. (S.12). The rest terms in Eq. (S.23) can be computed as

$$
\begin{aligned}
\frac{1}{2nN}\boldsymbol{\mu}(\mathcal{A}^{\mathrm{gn}})^{\intercal}\,\boldsymbol{\Sigma}^{-1}(\mathcal{A}^{\mathrm{gn}})\,\boldsymbol{\mu}(\mathcal{A}^{\mathrm{gn}}) &= \frac{\lambda^2 m^2}{2q} + o(1), \\
\frac{1}{nN}(\boldsymbol{y} \otimes \boldsymbol{1}_n)^{\intercal} \boldsymbol{I}_{n\beta}^2 (\boldsymbol{y} \otimes \boldsymbol{1}_n) &= \tau\beta(1-t_0) + (1-\tau)(-\beta t_1), \\
\frac{1}{nN} \times \text{last two lines in Eq. (S.23)} &= \tau \frac{(2\beta(1-t_0)q + \lambda m)^2}{2q(1+2\beta(1-t_0)q)} + (1-\tau)\frac{(2(-\beta t_1)q + \lambda m)^2}{2q(1+2(-\beta t_1)q)} + o(1).
\end{aligned}
$$

Collecting everything we get $G(\mathcal{A}^{\mathrm{gn}})$ in Eq. (S.12). Note that $G(\mathcal{A}^{\mathrm{gn}})/\beta = \mathcal{O}(1)$, and we are going to show that $G(\mathcal{A}^{\mathrm{gn}})/\beta - G(\mathcal{A}^{\mathrm{gs}})/\beta = o(1)$.

For $\mathcal{A}^{\mathrm{gs}}, \mathcal{A}^{\mathrm{bn}}$ and $\mathcal{A}^{\mathrm{bs}}$, we find the means and covariances of $(\boldsymbol{A} \otimes \boldsymbol{1}_n)\tilde{\boldsymbol{\sigma}}$ as

$$
\begin{aligned}
\boldsymbol{\mu}(\mathcal{A}^{\mathrm{gs}}) &= \boldsymbol{\mu}(\mathcal{A}^{\mathrm{gn}}), & \boldsymbol{\Sigma}(\mathcal{A}^{\mathrm{gs}}) &= \boldsymbol{\Sigma}(\mathcal{A}^{\mathrm{gn}}) + \frac{1}{N}\tilde{\boldsymbol{\sigma}}^{\intercal}\tilde{\boldsymbol{\sigma}}, \\
\boldsymbol{\mu}(\mathcal{A}^{\mathrm{bn}}) &= \boldsymbol{\mu}(\mathcal{A}^{\mathrm{gn}}) + \sqrt{d}\boldsymbol{1}_N \otimes \boldsymbol{l}, & \boldsymbol{\Sigma}(\mathcal{A}^{\mathrm{bn}}) &= \boldsymbol{\Sigma}(\mathcal{A}^{\mathrm{gn}})\left(1 + \mathcal{O}\left(\frac{1}{\sqrt{d}} + \frac{d}{N}\right)\right), \\
\boldsymbol{\mu}(\mathcal{A}^{\mathrm{bs}}) &= \boldsymbol{\mu}(\mathcal{A}^{\mathrm{gn}}) + \sqrt{d}\boldsymbol{1}_N \otimes \boldsymbol{l}, & \boldsymbol{\Sigma}(\mathcal{A}^{\mathrm{bs}}) &= \boldsymbol{\Sigma}(\mathcal{A}^{\mathrm{gn}})\left(1 + \mathcal{O}\left(\frac{1}{\sqrt{d}} + \frac{d}{N}\right)\right) + \frac{1 + \mathcal{O}\left(\frac{1}{\sqrt{d}} + \frac{d}{N}\right)}{N}\tilde{\boldsymbol{\sigma}}^{\intercal}\tilde{\boldsymbol{\sigma}},
\end{aligned}
\qquad [\text{S.26}]
$$

where $\boldsymbol{l} \in \mathbb{R}^n$ with entries $\boldsymbol{l}_a = \frac{\boldsymbol{1}_N^{\intercal}\boldsymbol{\sigma}^a}{N}$ are order parameters analogous to $\boldsymbol{m}$ in Eq. (S.6). Substituting Eq. (S.26) into Eq. (S.23), we see that the perturbation $\frac{1}{N}\tilde{\boldsymbol{\sigma}}^{\intercal}\tilde{\boldsymbol{\sigma}}$ in $\boldsymbol{\Sigma}(\mathcal{A}^{\mathrm{gs}})$ leads to a $\mathcal{O}(\frac{1}{N})$ perturbation in $G(\mathcal{A}^{\mathrm{gs}})$, while the perturbation of $\mathcal{O}\left(\frac{1}{\sqrt{d}} + \frac{d}{N}\right)$ in $\Sigma(\mathcal{A}^{\mathrm{bn}})$ and $\Sigma(\mathcal{A}^{\mathrm{bs}})$ leads to a $\mathcal{O}(\frac{1}{\sqrt{d}} + \frac{d}{N})$ perturbation in $G(\mathcal{A})$.

In $\boldsymbol{\mu}(\mathcal{A}^{\mathrm{bn}})$ and $\boldsymbol{\mu}(\mathcal{A}^{\mathrm{gn}})$, there is a bias $\boldsymbol{l}$. By the replica symmetric assumption Eq. (S.11), we have $\boldsymbol{l} = l\boldsymbol{1}_n$. It is easy to show that a critical point in the saddle point equation with l and $\hat{l}$ exists only when $l = 0$ for $\beta \to \infty$. Therefore, the term $\sqrt{d}\boldsymbol{1}_N \otimes \boldsymbol{l}$ will not influence the value of free energy density when $\beta \to \infty$. It further implies that the elements of $\boldsymbol{\sigma}^* = \boldsymbol{X}\boldsymbol{w}^*$ are symmetrically distributed around zero. This is analogous to the vanishing average magnetization for the Sherrington-Kirkpatrick model (1).

To summarize this section, as long as $\frac{1}{N}, \frac{1}{\sqrt{d}}, \frac{d}{N} \to 0$, averaging over $\mathcal{A}^{\mathrm{bs}}$, $\mathcal{A}^{\mathrm{gn}}$, $\mathcal{A}^{\mathrm{gs}}$ and $\mathcal{A}^{\mathrm{gn}}$ are equivalent in Eq. (S.16) when $\beta \to \infty$ for a one-layer GCN with $\boldsymbol{P}(\boldsymbol{A}) = \boldsymbol{A}$.[†]

[†] In general it does not hold that $G(\mathcal{A}^{\mathrm{gn}}) = G(\mathcal{A}^{\mathrm{bn}})$ nor that $G(\mathcal{A}^{\mathrm{gs}}) = G(\mathcal{A}^{\mathrm{bs}})$. The equivalence stems from the fact that $\boldsymbol{\sigma}^* = \boldsymbol{X}\boldsymbol{w}^*$ are symmetrically distributed, but for any fixed $\boldsymbol{\sigma}^*$ with $l = \boldsymbol{1}_N^{\intercal}\boldsymbol{\sigma}^* \neq 0$, we have $G(\mathcal{A}^{\mathrm{gn}}) = G(\mathcal{A}^{\mathrm{bn}}) + l^2\mathcal{O}(1)$ and $G(\mathcal{A}^{\mathrm{gs}}) = G(\mathcal{A}^{\mathrm{bs}}) + l^2\mathcal{O}(1)$.

C. Computation of E. We recall Eq. (S.10) and denote $\bar{\boldsymbol{Q}} = \widehat{\boldsymbol{Q}} + \operatorname{diag}(\widehat{\boldsymbol{Q}})$, then

$$\begin{aligned} E(\widehat{\boldsymbol{m}}, \widehat{\boldsymbol{Q}}) =& \frac{1}{nN} \ln \mathbb{E}_{\boldsymbol{X}} \sqrt{\frac{(2\pi)^{nN}}{\det\left(\boldsymbol{X}^\intercal \boldsymbol{X} \otimes \bar{\boldsymbol{Q}} + 2\tau r\beta \boldsymbol{I}_{nF}\right)}} \\ & \times \exp\left(\frac{1}{2}\left(\boldsymbol{y}^\intercal \boldsymbol{X} \otimes \widehat{\boldsymbol{M}}^\intercal\right)\left(\boldsymbol{X}^\intercal \boldsymbol{X} \otimes \bar{\boldsymbol{Q}} + 2r\tau\beta \boldsymbol{I}_{nF}\right)^{-1}\left(\boldsymbol{X}^\intercal \boldsymbol{y} \otimes \widehat{\boldsymbol{M}}\right)\right). \end{aligned} \tag{S.27}$$

We can compute the determinant term and the exponential term in Eq. (S.27) separately when $N \to \infty$. Denoting by $\lambda_f(\boldsymbol{X}^\intercal \boldsymbol{X})$ the f-th largest eigenvalue of $\boldsymbol{X}^\intercal \boldsymbol{X}$, we have

$$\begin{aligned} & \frac{1}{nN} \ln\left(\sqrt{\frac{(2\pi)^{nN}}{\det\left(\boldsymbol{X}^\intercal \boldsymbol{X} \otimes \widehat{\boldsymbol{Q}} + 2\tau r\beta \boldsymbol{I}_{nF}\right)}}\right) \\ =& -\frac{\widehat{p}}{2\gamma\left(2\widehat{q}+\widehat{p}\right)}\left(1 - \sum_{f}^{F} \frac{1}{F} \frac{2\tau r\beta/\left(2\widehat{q}+\widehat{p}\right)}{\lambda_f + 2\tau r\beta/\left(2\widehat{q}+\widehat{p}\right)}\right) + \frac{1}{2\gamma}\log\left(2\pi\right) - \frac{1}{F}\frac{n}{2\gamma}\sum_{f=1}^{F}\log\left(\left(2\widehat{q}+\widehat{p}\right)\lambda_f + 2\tau r\beta\right) + o(1). \end{aligned} \tag{S.28}$$

For the same reasons as in Eq. (S.25), the two logarithmic terms in the last line of Eq. (S.28) can be ignored since they do not grow with β. We denote $\widehat{r} \overset{\text{def}}{=} 2\tau r\beta/\left(2\widehat{q}+\widehat{p}\right)$. The first term in the RHS of Eq. (S.28) is then

$$\begin{aligned} T(\widehat{r}, \gamma) &= \frac{1}{\widehat{r}} \sum_{f}^{F} \frac{1}{F} \frac{\widehat{r}}{\lambda_f + \widehat{r}} \\ &= \frac{1}{2\widehat{r}}\left(1 - \widehat{r} + \sqrt{1 + \widehat{r}^2 + 2\widehat{r} + 2\gamma\widehat{r} - 2\gamma + \gamma^2} - \gamma\right) + o(1), \end{aligned} \tag{S.29}$$

which is obtained by integrating over the Marchenko–Pastur distribution.

The term inside the exponential in Eq. (S.27) can be computed as

$$\begin{aligned} & \frac{1}{2nN}\left(\boldsymbol{y}^\intercal \boldsymbol{X} \otimes \widehat{\boldsymbol{m}}^\intercal\right)\left(\boldsymbol{X}^\intercal \boldsymbol{X} \otimes \bar{\boldsymbol{Q}} + 2\tau r\beta \boldsymbol{I}_{nF}\right)^{-1}\left(\boldsymbol{X}^\intercal \boldsymbol{y} \otimes \widehat{\boldsymbol{m}}\right) \\ =& \frac{1}{2nN}\left(\boldsymbol{y}^\intercal \boldsymbol{X} \otimes \widehat{\boldsymbol{m}}^\intercal\right)\left(\boldsymbol{X}^\intercal \boldsymbol{X} \otimes \left(\bar{\boldsymbol{Q}} - p\boldsymbol{1}_n\boldsymbol{1}_n{}^\intercal\right) + 2\tau r\beta \boldsymbol{I}_{nF}\right)^{-1}\left(\boldsymbol{X}^\intercal \boldsymbol{y} \otimes \widehat{\boldsymbol{m}}\right) + o(1) \\ =& \frac{1}{2nN}\left(\widehat{\boldsymbol{m}}^\intercal\left(\bar{\boldsymbol{Q}} - \widehat{p}\boldsymbol{1}_n\boldsymbol{1}_n\right)^{-1}\widehat{\boldsymbol{m}}\right)\left(\boldsymbol{y}^\intercal \boldsymbol{X}\left(\boldsymbol{X}^\intercal \boldsymbol{X} + \widehat{r}\boldsymbol{I}_F\right)^{-1}\boldsymbol{X}^\intercal \boldsymbol{y}\right) + o(1) \\ =& \frac{1}{2}\frac{\widehat{m}^2}{2\widehat{q}+\widehat{p}}\left(1 - \frac{T\widehat{r} + \gamma - 1}{T^2\mu\widehat{r} + (\gamma-1)T\mu + \gamma}\right) + o(1), \end{aligned}$$

in which we first perturb $\bar{\boldsymbol{Q}}$ by $p\boldsymbol{1}_n\boldsymbol{1}_n^\intercal$ in the second line and then compute $\left(\boldsymbol{y}^\intercal \boldsymbol{X}\left(\boldsymbol{X}^\intercal \boldsymbol{X} + \widehat{r}\boldsymbol{I}_F\right)^{-1}\boldsymbol{X}^\intercal \boldsymbol{y}\right)$ by the Woodbury matrix identity.

2. Self-loop computation

When $\boldsymbol{P}(\boldsymbol{A}) = \boldsymbol{A} + c\boldsymbol{I}_N$, we still follow the replica pipeline from Section 1 but with different (more complicated) C and E in Eq. (S.9).

A. C with self-loop. We replace $\boldsymbol{A}$ by $\boldsymbol{A} + c\boldsymbol{I}_N$ in Eq. (S.7). Now the expectation over $\boldsymbol{A}$ depends not only on $\boldsymbol{m}$ and $\boldsymbol{Q}$, but also

$$\begin{aligned} \left(\boldsymbol{m}^0\right)_a &= \frac{1}{N}\boldsymbol{y}^\intercal \boldsymbol{I}_{\text{train}}\boldsymbol{\sigma}^a, & \left(\boldsymbol{m}^1\right)_a &= \frac{1}{N}\boldsymbol{y}^\intercal \boldsymbol{I}_{\text{test}}\boldsymbol{\sigma}^a, \\ \left(\boldsymbol{Q}^0\right)_{ab} &= \frac{1}{N}\left(\boldsymbol{\sigma}^a\right)^\intercal \boldsymbol{I}_{\text{train}}\boldsymbol{\sigma}^b, & \left(\boldsymbol{Q}^1\right)_{ab} &= \frac{1}{N}\left(\boldsymbol{\sigma}^a\right)^\intercal \boldsymbol{I}_{\text{test}}\boldsymbol{\sigma}^b. \end{aligned} \tag{S.30}$$

For $\boldsymbol{A} \sim \mathcal{A}^{\text{gn}}$, since $((\boldsymbol{A} + c\boldsymbol{I}_N) \otimes \boldsymbol{1}_n)\tilde{\boldsymbol{\sigma}}$ is a Gaussian with mean and covariance

$$\boldsymbol{\mu}(\mathcal{A}^{\text{gn}}) = \lambda\boldsymbol{y} \otimes \boldsymbol{m} + c\tilde{\boldsymbol{\sigma}}, \quad \boldsymbol{\Sigma}(\mathcal{A}^{\text{gn}}) = \boldsymbol{I}_N \otimes \boldsymbol{Q},$$

we can compute Eq. (S.23) directly. Leveraging the replica symmetric assumption, i.e.,

$$\begin{aligned} \boldsymbol{m}^0 &= m_0\boldsymbol{1}_n & \boldsymbol{m}^1 &= m_1\boldsymbol{1}_n \\ \boldsymbol{Q}^0 &= q_0\boldsymbol{I}_n + p_0\boldsymbol{1}_n\boldsymbol{1}_n^\intercal & \boldsymbol{Q}^1 &= q_1\boldsymbol{I}_n + p_1\boldsymbol{1}_n\boldsymbol{1}_n^\intercal, \end{aligned}$$

we have

$$C(\boldsymbol{m}^0,\boldsymbol{m}^1,\boldsymbol{Q}^0,\boldsymbol{Q}^1) = \tau\frac{(2\beta_0 q+\lambda m)^2}{q\,(1+2\beta_0 q)} + (1-\tau)\,\frac{(2\beta_1 q+\lambda m)^2}{q\,(1+2\beta_1 q)}$$
$$+\, c^2\frac{(1+2\beta_0 q)\,p_0 q-(1+4\beta_0 q)\,pq_0}{q^2\,(1+2\beta_0 q)^2} + c^2\frac{(1+2\beta_1 q)\,p_1 q-(1+4\beta_1 q)\,pq_1}{q^2\,(1+2\beta_1 q)^2}$$
$$+\, 2c\left(\frac{\lambda m+2\beta_0 q}{q\,(1+2\beta_0 q)}m_0+\frac{\lambda m+2\beta_1 q}{q\,(1+2\beta_1 q)}m_1\right)+o(1)+o_\beta(1).$$

where $\beta_0=\beta(1-t_0)$, $\beta_1=-\beta t_1$, $q=q_0+q_1$, $m=m_0+m_1$ and $p=p_0+p_1$.

B. E **with self-loop.** After applying the Fourier representation method Eq. (S.8) to the order parameters Eq. (S.30), we obtain dual variables $\widehat{\boldsymbol{m}}^0,\widehat{\boldsymbol{m}}^1,\widehat{\boldsymbol{Q}}^0,\widehat{\boldsymbol{Q}}^1$. We then get a new Eq. (S.10) as

$$E(\widehat{\boldsymbol{m}}^0,\widehat{\boldsymbol{m}}^1,\widehat{\boldsymbol{Q}}^0,\widehat{\boldsymbol{Q}}^1)=\frac{1}{nN}\ln\mathbb{E}_{\boldsymbol{X}}\int\prod_{a=1}^{n}\mathrm{d}\boldsymbol{w}_a\exp\Bigg(-\sum_{a\le b}(\widehat{\boldsymbol{Q}}^0)_{ab}\,(\boldsymbol{w}^a)^\intercal\,\boldsymbol{X}^\intercal\boldsymbol{I}_{\text{train}}\boldsymbol{X}w^b-\sum_a(\widehat{\boldsymbol{m}}^0)_a\boldsymbol{y}^\intercal\boldsymbol{X}\boldsymbol{I}_{\text{train}}\boldsymbol{w}^a$$
$$-\sum_{a\le b}(\widehat{\boldsymbol{Q}}^1)_{ab}\,(\boldsymbol{w}^a)^\intercal\,\boldsymbol{X}^\intercal\boldsymbol{I}_{\text{test}}\boldsymbol{X}\boldsymbol{w}^b-\sum_a(\widehat{\boldsymbol{m}}^1)_a\boldsymbol{y}^\intercal\boldsymbol{X}\boldsymbol{I}_{\text{test}}\boldsymbol{w}^a-\tau r\beta\sum_a\|\boldsymbol{w}^a\|_2^2\Bigg).$$

Integrating over $\boldsymbol{w}_a$ yields

$$E=\frac{1}{nN}\ln\mathbb{E}_{\boldsymbol{X}}\sqrt{\frac{(2\pi)^{nN}}{\det\left(\boldsymbol{X}^\intercal\boldsymbol{I}_{\text{train}}\boldsymbol{X}\otimes\bar{\boldsymbol{Q}}^0+\boldsymbol{X}^\intercal\boldsymbol{I}_{\text{test}}X\otimes\bar{\boldsymbol{Q}}^1+2\tau r\beta\boldsymbol{I}_{nF}\right)}}$$
$$\times\exp\left(\frac{1}{2}\left(\boldsymbol{y}^\intercal\otimes\left(\widehat{\boldsymbol{m}}^0\right)^\intercal\boldsymbol{I}_{\text{train}}\boldsymbol{X}+\boldsymbol{y}^\intercal\boldsymbol{I}_{\text{test}}\boldsymbol{X}\otimes\left(\widehat{\boldsymbol{m}}^1\right)^\intercal\right)\right.$$
$$\left.\times\left(\boldsymbol{X}^\intercal I_{\text{train}}\boldsymbol{X}\otimes\bar{\boldsymbol{Q}}^0+\boldsymbol{X}^\intercal\boldsymbol{I}_{\text{test}}\boldsymbol{X}\otimes\bar{\boldsymbol{Q}}^1+2\tau r\beta\boldsymbol{I}_{nF}\right)^{-1}\left(\boldsymbol{X}^\intercal\boldsymbol{I}_{\text{train}}\boldsymbol{y}\otimes\widehat{\boldsymbol{m}}^0+\boldsymbol{X}^\intercal\boldsymbol{I}_{\text{test}}\boldsymbol{y}\otimes\widehat{\boldsymbol{m}}^1\right)\right),\qquad\text{[S.31]}$$

where $\bar{\boldsymbol{Q}}^0=\widehat{\boldsymbol{Q}}^0+\operatorname{diag}(\widehat{\boldsymbol{Q}}^0)$ and $\bar{\boldsymbol{Q}}^1=\widehat{\boldsymbol{Q}}^1+\operatorname{diag}(\widehat{\boldsymbol{Q}}^1)$. By replica symmetry, we have

$$\widehat{\boldsymbol{m}}^0=\widehat{m}_0\boldsymbol{1}_n\qquad\widehat{\boldsymbol{m}}^1=\widehat{m}_1\boldsymbol{1}_n$$
$$\bar{\boldsymbol{Q}}_0=\widehat{q}_0\boldsymbol{I}_n+\widehat{p}_0\boldsymbol{1}_n\boldsymbol{1}_n^\intercal\qquad\bar{\boldsymbol{Q}}_1=\widehat{q}_1\boldsymbol{I}+\widehat{p}_1\boldsymbol{1}_n\boldsymbol{1}_n^\intercal.\qquad\text{[S.32]}$$

Similarly as in Appendix C, we can compute the determinant term and the exponential term separately when $N\to\infty$. For the sake of simplicity, we only consider $r=0$ and $\mu=0$ in what follows. Denoting $\boldsymbol{Y}^0=\boldsymbol{X}^\intercal\boldsymbol{I}_{\text{train}}\boldsymbol{X}$ and $\boldsymbol{Y}^1=\boldsymbol{X}^\intercal\boldsymbol{I}_{\text{test}}\boldsymbol{X}$, we write the determinant term in Eq. (S.31) in the $n\to0$ limit as

$$\lim_{n\to0}\frac{1}{nN}\ln\det\left(\boldsymbol{Y}^0\otimes\bar{\boldsymbol{Q}}^0+\boldsymbol{Y}^1\otimes\bar{\boldsymbol{Q}}^1\right)$$
$$=\lim_{n\to0}\frac{1}{nN}\ln\left(\boldsymbol{I}_F+n\left(\widehat{p}_0\boldsymbol{Y}^0+\widehat{p}_1\boldsymbol{Y}^1\right)\left(\widehat{q}_0\boldsymbol{Y}^0+\widehat{q}_1\boldsymbol{Y}^1\right)^{-1}\right)\left(\widehat{q}_0\boldsymbol{Y}^0+\widehat{q}_1\boldsymbol{Y}^1\right)^n\qquad\text{[S.33]}$$
$$=\frac{1}{N}\operatorname{Tr}\left(\widehat{p}_0\boldsymbol{Y}^0+\widehat{p}_1\boldsymbol{Y}^1\right)\left(\widehat{q}_0\boldsymbol{Y}^0+\widehat{q}_1\boldsymbol{Y}^1\right)^{-1}.$$

The exponential term in Eq. (S.31) can be computed similarly, with noticing that $\boldsymbol{Y}^0$ and $\boldsymbol{Y}^1$ are rotationally invariant since $\mu=0$,

$$\exp\left\{\frac{1}{2}\left(\boldsymbol{y}^\intercal\boldsymbol{I}_{\text{train}}\boldsymbol{X}\otimes\left(\widehat{\boldsymbol{m}}^0\right)^\intercal+\boldsymbol{y}^\intercal\boldsymbol{I}_{\text{test}}\boldsymbol{X}\otimes\left(\widehat{\boldsymbol{m}}^1\right)^\intercal\right)\left(\boldsymbol{X}^\intercal\boldsymbol{I}_{\text{train}}\boldsymbol{X}\otimes\bar{\boldsymbol{Q}}^0+\boldsymbol{X}^\intercal\boldsymbol{I}_{\text{test}}\boldsymbol{X}\otimes\bar{\boldsymbol{Q}}^1\right)^{-1}\right.$$
$$\left.\times\left(\boldsymbol{X}^\intercal\boldsymbol{I}_{\text{train}}\boldsymbol{y}\otimes\widehat{\boldsymbol{m}}^0+\boldsymbol{X}^\intercal\boldsymbol{I}_{\text{test}}\boldsymbol{y}\otimes\widehat{\boldsymbol{m}}^1\right)\right\}\qquad\text{[S.34]}$$
$$=\exp\left\{\operatorname{Tr}\left(n\left(\widehat{q}_0\boldsymbol{Y}^0+\widehat{p}_1\boldsymbol{Y}^1\right)\left(\widehat{m}_0^2\boldsymbol{Y}^0+\widehat{m}_1^2\boldsymbol{Y}^1\right)^{-1}\right)\right\},$$

Both Eq. (S.33) and Eq. (S.34) involve the same quantity,

$$U(a,b,c,d)\overset{\text{def}}{=}\mathbb{E}_{\boldsymbol{X}}\left[\operatorname{Tr}\left(\left(a\boldsymbol{Y}^0+b\boldsymbol{Y}^1\right)\left(c\boldsymbol{Y}^0+d\boldsymbol{Y}^1\right)^{-1}\right)\right]$$
$$=\frac{a}{c}\mathbb{E}_{\boldsymbol{X}}\left[\operatorname{Tr}\left(\boldsymbol{Y}^0\left(\boldsymbol{Y}^0+\frac{d}{c}\boldsymbol{Y}^1\right)^{-1}\right)\right]+\frac{b}{d}\mathbb{E}_{\boldsymbol{X}}\left[\operatorname{Tr}\left(\boldsymbol{Y}^1\left(\frac{c}{d}\boldsymbol{Y}^0+\boldsymbol{Y}^1\right)^{-1}\right)\right],\qquad\text{[S.35]}$$

which can be computed by random matrix free convolution (2). The Green's function (also called the Cauchy function in the mathematical literature) is defined via the Stieltjes transform

$$G_{\boldsymbol{Y}}(z) := \int \frac{\rho_{\boldsymbol{Y}}(\lambda)}{z-\lambda} \mathrm{d}\lambda = \frac{1}{N} \operatorname{Tr}\left(z \boldsymbol{I}_N - \boldsymbol{Y}\right)^{-1}, \tag{S.36}$$

which in turn yields the spectrum transform $\rho_{\boldsymbol{Y}}(\lambda) = -\frac{1}{\pi} \lim_{\epsilon \to 0} \Im G_{\boldsymbol{Y}}(z)\big|_{z=\lambda+i\epsilon}$. The corresponding Voiculescu S–transform reads

$$S_{\boldsymbol{Y}}(w) = \frac{1+w}{w}\chi(w) \quad \text{where } \frac{1}{\chi(w)} G_{\boldsymbol{Y}}\left(\frac{1}{\chi(w)}\right) - 1 = w \text{ and } \frac{1}{\chi(w)} = z.$$

We get the multiplicative free convolution as

$$S_{\boldsymbol{Y}^0\boldsymbol{Y}^1}(w) = S_{\boldsymbol{Y}^0}(w) S_{\boldsymbol{Y}^1}(w). \tag{S.37}$$

After computing $S_{\boldsymbol{Y}^0(\boldsymbol{Y}^1)^{-1}}$ and $S_{\boldsymbol{Y}^1(\boldsymbol{Y}^0)^{-1}}$, we obtain the expression for U in Eq. (S.35). For example, when $\tau = 0.8$ and $\gamma = 5$, the eigenvalue distributions of $\boldsymbol{Y}_0, \boldsymbol{Y}_1$ asymptotically follow the Marchenko–Pastur distribution as

$$\rho_{\boldsymbol{Y}^0}(\lambda) = \frac{\sqrt{16-\lambda}}{8\pi\sqrt{\lambda}} \mathbb{1}_{\lambda \in \{1,9\}}, \quad \rho_{\boldsymbol{Y}^1}(\lambda) \quad = \frac{\sqrt{4-\lambda}}{2\pi\sqrt{\lambda}} \mathbb{1}_{\lambda \in \{0,1\}}.$$

We then get

$$U(a,b,c,d) = -\frac{aF}{c} \frac{-\frac{2d}{c} + \sqrt{9 + \frac{16d}{c}} - 3}{2\frac{d}{c}(\frac{d}{c} - 1)} - \frac{bF}{d} \frac{5 - \sqrt{\frac{\frac{9c}{d}+16}{\frac{c}{d}}}}{-\frac{2c}{d} + 2}. \tag{S.38}$$

Once C and E are computed, we have all the ingredients of the saddle point equation Eq. (S.16) with 12 variables,

$$m_0,\ m_1,\ \widehat{m_0},\ \widehat{m_1},\ p_0,\ p_1,\ \widehat{p_0},\ \widehat{p_1},\ q_0,\ q_1,\ \widehat{q_0} \text{ and } \widehat{m_1}.$$

A critical point of this saddle point equation gives the explicit formulas for the risks in Section 1.

3. A random matrix theory approach

As mentioned in the main paper, if we start with the Gaussian adjacency matrices defined before Conjecture 1 we can obtain some of the results described above. For simplicity, we outline this approach for the full observation case $\boldsymbol{I}_{\text{train}} = \boldsymbol{I}_N$, that is, for $\tau = 1$, and compute the empirical risk. The partial observation case follows the same strategy but involves more complicated calculations. We let $\alpha \stackrel{\text{def}}{=} \frac{1}{\gamma} = \frac{F}{N}$, and rescale variables as $\sqrt{\mu\gamma} \to \mu$, $\sqrt{F}u \to u$. Following Conjecture 1, we replace the binary symmetric adjacency matrix $\boldsymbol{A}^{\text{bs}}$ by the Gaussian random matrix with a rank-one spike so that

$$\boldsymbol{A} = \boldsymbol{\Xi}^{\text{gn}} + \frac{\lambda}{N}\boldsymbol{y}\boldsymbol{y}^{\mathsf{T}},$$
$$\boldsymbol{X} = \boldsymbol{\Xi}^{x} + \frac{\mu}{N}\boldsymbol{y}\boldsymbol{u}^{\mathsf{T}}.$$

The ridge loss reads

$$L(\boldsymbol{w}) = \frac{1}{N}\|\boldsymbol{y} - \boldsymbol{A}\boldsymbol{X}\boldsymbol{w}\|_2^2 + \frac{r}{N}\|\boldsymbol{w}\|^2,$$

and has the unique minimum

$$\begin{aligned}
\boldsymbol{w}^* &= \arg\min_{\boldsymbol{w}} L(\boldsymbol{w}) \\
&= \left(r\boldsymbol{I}_F + \boldsymbol{X}^{\mathsf{T}}\boldsymbol{A}^{\mathsf{T}}\boldsymbol{A}\boldsymbol{X}\right)^{-1}\boldsymbol{X}^{\mathsf{T}}\boldsymbol{A}^{\mathsf{T}} \\
&= \left(r\boldsymbol{I}_F + \boldsymbol{\Phi}^{\mathsf{T}}\boldsymbol{\Phi}\right)^{-1}\boldsymbol{\Phi}^{\mathsf{T}}\boldsymbol{y},
\end{aligned}$$

with $\boldsymbol{\Phi} = \boldsymbol{A}\boldsymbol{X}$. We need to compute the empirical risk,

$$\begin{aligned}
R_{\text{train}} &= \frac{1}{N}\|\boldsymbol{y} - \boldsymbol{A}\boldsymbol{X}\boldsymbol{w}^*\|_2^2 \\
&= \frac{r^2}{N}\operatorname{Tr}\left(\boldsymbol{y}\boldsymbol{y}^{\mathsf{T}}\boldsymbol{Q}^2\right) \\
&= -\frac{r^2}{N}\frac{\partial}{\partial r}\operatorname{Tr}\left(\boldsymbol{y}\boldsymbol{y}^{\mathsf{T}}\boldsymbol{Q}\right),
\end{aligned}$$

as well as the empirical loss

$$L(\boldsymbol{w}^*) = r\boldsymbol{y}^{\mathsf{T}}\boldsymbol{Q}\boldsymbol{y}/N, \tag{S.39}$$

where we set $\boldsymbol{Q} \overset{\text{def}}{=} (r\boldsymbol{I}_N + \boldsymbol{\Phi}\boldsymbol{\Phi}^\intercal)^{-1}$.

We first define four thin matrices,

$$
\begin{aligned}
\boldsymbol{U} &= \begin{bmatrix} \frac{1}{\sqrt{N}}\boldsymbol{\Xi}^{\text{gn}}\boldsymbol{y} + \frac{\lambda}{\sqrt{N}}\boldsymbol{y} & \frac{\lambda}{\sqrt{N}}\boldsymbol{y} \end{bmatrix} \in \mathbb{R}^{N\times 2} \\
\boldsymbol{V} &= \begin{bmatrix} \frac{\mu}{\sqrt{N}}\boldsymbol{u} & \frac{1}{\sqrt{N}}\left(\boldsymbol{\Xi}^{x}\right)^\intercal \boldsymbol{y} \end{bmatrix} \in \mathbb{R}^{F\times 2}, \\
\boldsymbol{L} &= \begin{bmatrix} \boldsymbol{O}\boldsymbol{V} + \boldsymbol{U}\boldsymbol{V}^\intercal\boldsymbol{V} & \boldsymbol{U} \end{bmatrix} \in \mathbb{R}^{N\times 4}, \\
\boldsymbol{M} &= \begin{bmatrix} \boldsymbol{U} & \boldsymbol{O}\boldsymbol{V} \end{bmatrix} \in \mathbb{R}^{F\times 4}.
\end{aligned} \qquad \text{[S.40]}
$$

where $\boldsymbol{O} = \boldsymbol{\Xi}^{\text{gn}}\boldsymbol{\Xi}^{x}$. Then

$$
\begin{aligned}
\boldsymbol{\Phi} &= \boldsymbol{U}\boldsymbol{V}^\intercal + \boldsymbol{O} \\
\boldsymbol{\Phi}\boldsymbol{\Phi}^\intercal &= \boldsymbol{L}\boldsymbol{M}^\intercal + \boldsymbol{O}\boldsymbol{O}^\intercal
\end{aligned} \qquad \text{[S.41]}
$$

Using Woodbury matrix identity, and denoting $\boldsymbol{R} = (r\boldsymbol{I}_N + \boldsymbol{O}\boldsymbol{O}^\intercal)^{-1}$, we have

$$
\begin{aligned}
\boldsymbol{Q} &= (r\boldsymbol{I}_N + \boldsymbol{O}\boldsymbol{O}^\intercal + \boldsymbol{L}\boldsymbol{M}^\intercal)^{-1} \\
&= (r\boldsymbol{I}_N + \boldsymbol{O}\boldsymbol{O}^\intercal)^{-1} - (r\boldsymbol{I}_N + \boldsymbol{O}\boldsymbol{O}^\intercal)^{-1}\boldsymbol{L}\left(\boldsymbol{I}_4 + \boldsymbol{M}^\intercal(r\boldsymbol{I}_N + \boldsymbol{O}\boldsymbol{O}^\intercal)^{-1}\boldsymbol{L}\right)^{-1}\boldsymbol{M}^\intercal(r\boldsymbol{I}_N + \boldsymbol{O}\boldsymbol{O}^\intercal)^{-1} \\
&= \boldsymbol{R} - \boldsymbol{R}\boldsymbol{L}\left(\boldsymbol{I}_N + \boldsymbol{M}^\intercal\boldsymbol{R}\boldsymbol{L}\right)^{-1}\boldsymbol{M}^\intercal\boldsymbol{R}.
\end{aligned}
$$

Now Eq. (S.39) can be computed as

$$
\frac{1}{N}\boldsymbol{y}^\intercal\boldsymbol{Q}\boldsymbol{y} = \underbrace{\frac{1}{N}\boldsymbol{y}^\intercal\boldsymbol{R}\boldsymbol{y}}_{\mathbb{R}^{1\times 1}} - \underbrace{\frac{1}{\sqrt{N}}\boldsymbol{y}^\intercal\boldsymbol{R}\boldsymbol{L}}_{\mathbb{R}^{1\times 4}}\underbrace{\left(\boldsymbol{I}_4 + \boldsymbol{M}^\intercal\boldsymbol{R}\boldsymbol{L}\right)^{-1}}_{\mathbb{R}^{4\times 4}}\underbrace{\frac{1}{\sqrt{N}}\boldsymbol{M}^\intercal\boldsymbol{R}\boldsymbol{y}}_{\mathbb{R}^{4\times 1}}. \qquad \text{[S.42]}
$$

The curly braces indicate 25 random variables which all concentrate around their means (they are self-averaging in statistical physics terminology). Their expectations can be computed as follows:

- $\frac{1}{N}\boldsymbol{y}^\intercal\boldsymbol{R}\boldsymbol{y}$: The first term of the RHS of S.42 is a special case discussed in Section 3.2.1 (3) and also has been discussed in (4). Recalling $\boldsymbol{R} = (r\boldsymbol{I}_N + \boldsymbol{O}\boldsymbol{O}^\intercal)^{-1}$, we first compute the Green function S.36 of $\boldsymbol{O}\boldsymbol{O}^\intercal$ as

$$
G_{\boldsymbol{O}\boldsymbol{O}^\intercal}(z) = \frac{1}{z}P\left(\frac{1}{z}\right)
$$

  where $P(t)$ is the solution of

$$
P = 1 + \frac{(1 + (P-1)/\alpha)(1 + (P-1))\,t}{1 - (1 + (P-1)/\alpha)(1 + (P-1))\,Pt}. \qquad \text{[S.43]}
$$

  Since $\boldsymbol{R}$ is rotationally invariant, we have

$$
\mathbb{E}\,\frac{1}{N}\boldsymbol{y}^\intercal\boldsymbol{R}\boldsymbol{y} = \mathbb{E}\,\frac{1}{N}\mathrm{Tr}(\boldsymbol{y}\boldsymbol{y}^\intercal\boldsymbol{R}) = \mathbb{E}\,\frac{1}{N}\mathrm{Tr}(\boldsymbol{R}) = q,
$$

  where q is the real solution of

$$
qr = 1 - \frac{(1 + (qr-1)/\alpha)\,q}{1 + (1 + (qr-1)/\alpha)\,q}.
$$

  It is easy to check that when $r \to 0$, we have $q \to \frac{1-\alpha}{r}$ for $\alpha \leq 1$.

- $\frac{1}{\sqrt{N}}\boldsymbol{y}^\intercal\boldsymbol{R}\boldsymbol{L}$ and $\frac{1}{\sqrt{N}}\boldsymbol{M}^\intercal\boldsymbol{R}\boldsymbol{y}$: we use Eq. (S.40) and recall the definition of $\boldsymbol{R}$ again we get

$$
\mathbb{E}\,\frac{1}{\sqrt{N}}\boldsymbol{y}^\intercal\boldsymbol{R}\boldsymbol{L} = \begin{bmatrix} \alpha\mu^2\lambda & \lambda & \lambda & \lambda \end{bmatrix} \times q,
$$

  as well as

$$
\mathbb{E}\,\frac{1}{\sqrt{N}}\boldsymbol{M}^\intercal\boldsymbol{R}\boldsymbol{y} = \mathbb{E}\frac{1}{\sqrt{N}}\begin{bmatrix} \boldsymbol{U}^\intercal \\ \boldsymbol{V}^\intercal\boldsymbol{O}^\intercal \end{bmatrix}\boldsymbol{R}\boldsymbol{y} = \begin{bmatrix} \lambda \\ \lambda \\ 0 \\ 0 \end{bmatrix} q.
$$

- $(\boldsymbol{I}_4 + \boldsymbol{M}^\intercal \boldsymbol{R}\boldsymbol{L})^{-1}$: we find the entries of $\boldsymbol{M}^\intercal \boldsymbol{R}\boldsymbol{L}$ are self-averaging, and we again use Eq. (S.40) to average $\boldsymbol{M}^\intercal \boldsymbol{R}\boldsymbol{L}$,

$$\mathbb{E}\ \boldsymbol{M}^\intercal \boldsymbol{R}\boldsymbol{L} = \begin{bmatrix} \alpha\mu^2 a + \alpha\lambda^2\mu^2 q & b+\lambda^2 q & a+\lambda^2 q & \lambda^2 q \\ \alpha\lambda^2\mu^2 q & \lambda^2 q & \lambda^2 q & \lambda^2 q \\ \mu^2 c & 0 & 0 & 0 \\ \alpha\mu^2 b & d & b & 0 \end{bmatrix},$$

where

$$\begin{aligned} a(\alpha, r) &= \mathbb{E}\ \frac{1}{N}\boldsymbol{y}^\intercal \left(\boldsymbol{\Xi}^{\mathrm{gn}}\right)^\intercal \boldsymbol{R}\boldsymbol{\Xi}^{\mathrm{gn}}\boldsymbol{y} \\ b(\alpha, r) &= \mathbb{E}\ \frac{1}{N}\boldsymbol{y}^\intercal \boldsymbol{\Xi}^{x}\boldsymbol{O}^\intercal \boldsymbol{R}\boldsymbol{\Xi}^{\mathrm{gn}}\boldsymbol{y} \\ c(\alpha, r) &= \mathbb{E}\ \frac{1}{N}\boldsymbol{u}^\intercal \boldsymbol{O}^\intercal \boldsymbol{R}\boldsymbol{O}\boldsymbol{u} \\ d(\alpha, r) &= \mathbb{E}\ \frac{1}{N}\boldsymbol{y}^\intercal \boldsymbol{R}\boldsymbol{\Xi}^{x}\boldsymbol{O}^\intercal \boldsymbol{R}\boldsymbol{O}\boldsymbol{\Xi}^{x\intercal}\boldsymbol{y} \\ q(\alpha, r) &= \mathbb{E}\ \frac{1}{N}\boldsymbol{y}^\intercal \boldsymbol{R}\boldsymbol{y}. \end{aligned} \tag{S.44}$$

Now we have all the ingredients in the RHS of Eq. (S.42)/Eq. (S.39). Putting them together gives

$$\begin{aligned} \boldsymbol{L}(\boldsymbol{w}^*) &= \frac{r}{N}\left(\boldsymbol{y}^\intercal \boldsymbol{R}\boldsymbol{L} - \boldsymbol{y}^\intercal \boldsymbol{R}\boldsymbol{L}\left(\boldsymbol{I}_4 + \boldsymbol{M}^\intercal \boldsymbol{R}\boldsymbol{L}\right)^{-1}\boldsymbol{M}^\intercal \boldsymbol{R}\boldsymbol{y}\right) \\ &\xrightarrow{N\to\infty} r\left(q - q^2\left(\frac{\lambda^2\left(a\alpha\mu^2+c\mu^2\left(-\left(a+(b-1)^2\right)\right)+d\left(a\mu^2(c-\alpha)-1\right)+\alpha\mu^2+\alpha b^2\mu^2-2\alpha b\mu^2+1\right)}{\lambda^2 q\left(a\alpha\mu^2+c\mu^2\left(-\left(a+(b-1)^2\right)\right)+d\left(a\mu^2(c-\alpha)-1\right)+\alpha\mu^2+\alpha b^2\mu^2-2\alpha b\mu^2+1\right)+a\mu^2(\alpha-c)+1}\right)\right). \end{aligned} \tag{S.45}$$

The full expressions for quantities in Eq. (S.44) are complicated. We thus analytically study the ridgeless limit $r \to 0$ in which the following hold:

$$\begin{aligned} a &\to \frac{(1-\alpha)^2}{r}, \\ b &\to \alpha - \frac{\alpha^2}{(1-\alpha)^2}r, \\ c &\to \alpha - \frac{\alpha^2}{(1-\alpha)^2}r, \\ d &\to 1 - \frac{\alpha}{1-\alpha}r, \\ q &\to \frac{1-\alpha}{r} + \frac{\alpha^2}{(1-\alpha)^2}. \end{aligned}$$

Substituting into Eq. (S.45) yields

$$L(\boldsymbol{w}^*) \to \frac{(1-\alpha)\left(\alpha^2\mu^2+1\right)}{\alpha^2\left(\lambda^2+1\right)\mu^2+\alpha\lambda^2+1}.$$

Finally, reverse the rescaling of $\alpha \to \frac{1}{\gamma}$, $\mu^2 \to \mu\gamma$, we get the same expressions for R_{train} as in Eq. (18) for $\tau = 1$.

If we assume Conjecture 1 and begin with Gaussian adjacency matrices, this approach can be easily extended to multi-hop by defining $\boldsymbol{O} = \boldsymbol{P}(\Xi^{gn})\Xi^{x}$ and computing corresponding $\boldsymbol{L}, \boldsymbol{M}, \boldsymbol{R}$. We can then obtain a closed-form expression via Eq. (S.45) after a longer computation. For example, when $\boldsymbol{P}(\boldsymbol{A}) = \boldsymbol{A}^2$ (two hops), $\tau = 1, \mu = 0, r \to 0$, we get the training loss as

$$L(\boldsymbol{w}^*) \to \frac{(1-\alpha)\left(\alpha\lambda^2+1\right)}{\alpha\left(\lambda^2+2\right)\lambda^2+1}.$$

The accurate matching between the numerical and theoretical results in Figure S1 also supports Conjecture 1.

4. A signal processing interpretation of self-loops

We now show a simple interpretation of negative self-loops based on a graph signal processing intuition (5, 6). In homophilic graphs the labels change slowly on the graph: they are a low-pass signal (6, 7) with most of their energy concentrated on the eigenvectors of the graph Laplacian which correspond to small eigenvalues or small "frequencies". Equivalently, they correspond to large eigenvalues of the adjacency matrix since $\boldsymbol{L} = \mathrm{diag}(\boldsymbol{A}\boldsymbol{1}) - \boldsymbol{A}$.[‡] On heterophilic graphs the labels usually change across an edge, which corresponds to a high-frequency signal concentrated on the small-eigenvalue eigenvectors of the adjacency matrix. A graph Fourier transform can be defined via the Laplacian but also via the adjacency matrix (6). The matrix product $\boldsymbol{A}\boldsymbol{x} = \boldsymbol{h}$ is a delay-like filter, diagonal in the graph Fourier domain with basis functions which are the eigenvectors $\boldsymbol{u}_1, \ldots, \boldsymbol{u}_N$ of $\boldsymbol{A}$. We have $(\widehat{\boldsymbol{A}\boldsymbol{x}})_i = \langle \boldsymbol{x}, \boldsymbol{u}_i \rangle = \lambda_i \widehat{\boldsymbol{x}}_i = \lambda_i \langle \boldsymbol{x}, \boldsymbol{u}_i \rangle$, where λ_i is the i-th smallest eigenvalue of $\boldsymbol{A}$.

[‡] If node degrees are all the same the eigenvectors of the adjacency matrix and the Laplacian coincide.

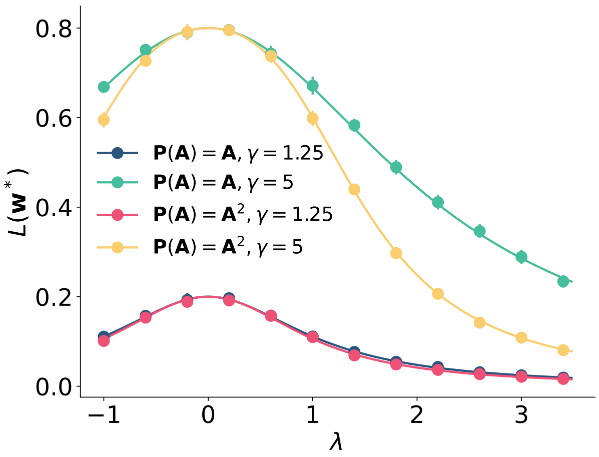

Fig. S1. Theoretical results (solid line) vs. experimental results (solid circles) for varying homophily of graphs (λ). We compare the one-hop case ($P(\boldsymbol{A}) = \boldsymbol{A}$) and two-hops case ($P(\boldsymbol{A}) = \boldsymbol{A}^2$) for non-symmetric CSBM with $\mu = 0, \tau = 1, N = 2000$ and $d = 30$. We use non-symmetric binary adjacency matrix $\mathcal{A}^{\text{bn}}$. Each experimental data point is averaged over 10 independent trials and the standard deviation is indicated by vertical lines.

Figure S2 illustrates the spectra of homophilic and heterophilic labels and graphs. A homophilic graph[§] has a low-pass spectrum while a heterophilic graph has a high-pass spectrum. A self-loop shifts the spectrum of $\boldsymbol{A}$ so that it becomes either a lowpass filter for positive c or a highpass filter for negative c. As a result, the corresponding GCNs better suppress noise and enhance signal for the corresponding graph types. In particular, assuming that the label-related signal in $\boldsymbol{x}$ lives between eigenvalues λ_a and λ_b (say, negative, so we are in a heterophilic situation), we can quantify the distortion induced by the filter $\boldsymbol{A} + c\boldsymbol{I}$ as $(\lambda_a + c)/(\lambda_b + c)$ which is close to 1 for large $|c|$.

5. Double descent in various GNNs

In Figure S3 we experiment with node classification on the `citeeer` dataset and some popular GNN architectures: the graph attention network (8), GraphSAGE (9), and Chebyshev graph network (10). The architectures of these GNNs incorporate various strategies to mitigate overfitting. As a result there is no clear double descent in the test accuracy curves, but we still observe non-monotonicity in the test risk.

6. Experimental Details

In this section we provide more details for the experiments in the main text.

In the real-world data experiments, all GCNs in Figure 1 are trained by the ADAM optimizer with learning rate 10^{-2} and weight decay 10^{-5}. We run ADAM for 10^4 iterations and select the model with minimal training loss. In each trial, the training and test nodes are selected uniformly randomly. We sample training nodes separately for each label to avoid the pathology where a label has few or zero samples, which can happen at extremely low training ratios. We average 10 different trials for each point; the error bars show their standard deviation. The standard deviation in the figures is mainly due to the train-test splits in the different trials; The fluctuations due to random initialization and stochastic optimization training are comparatively small. We do not normalize features and reprocess the data. All results in this paper are fully reproducible; code available at https://github.com/DaDaCheng/SMGCN.

Datasets	Cora	Citeseer	Squirrel	Chameleon	Texas
Features (F)	1433	3703	2089	2325	1703
Nodes (N)	2708	3327	5201	2277	183
Edges	5278	4552	198353	31371	279
Inverse relative model complexity ($\gamma = N/F$)	1.89	0.90	2.49	0.98	0.11
$H(G)$	0.825	0.718	0.217	0.247	0.057

Table S1. Benchmark dataset properties and statistics. $H(G)$ **is the level of homophily defined in (11).**

For the CSBM experiments in Fig. 3,5 and 6, we calculate $\boldsymbol{w}^*$ by Eq. (11) and then compute Eq. (5) and Eq. (5). In Fig. 3 and 5, we use symmetric binary adjacency matrix set $\mathcal{A}^{\text{bs}}$; In Fig. 6 we use non-symmetric binary adjacency matrix $\mathcal{A}^{\text{bn}}$ as defined in Conjecture 1. The theoretical results in Fig. 3, 4,5 and 6 are obtained by computing the extreme values in S.18.

[§] More precisely, a homophilic graph–label pair.

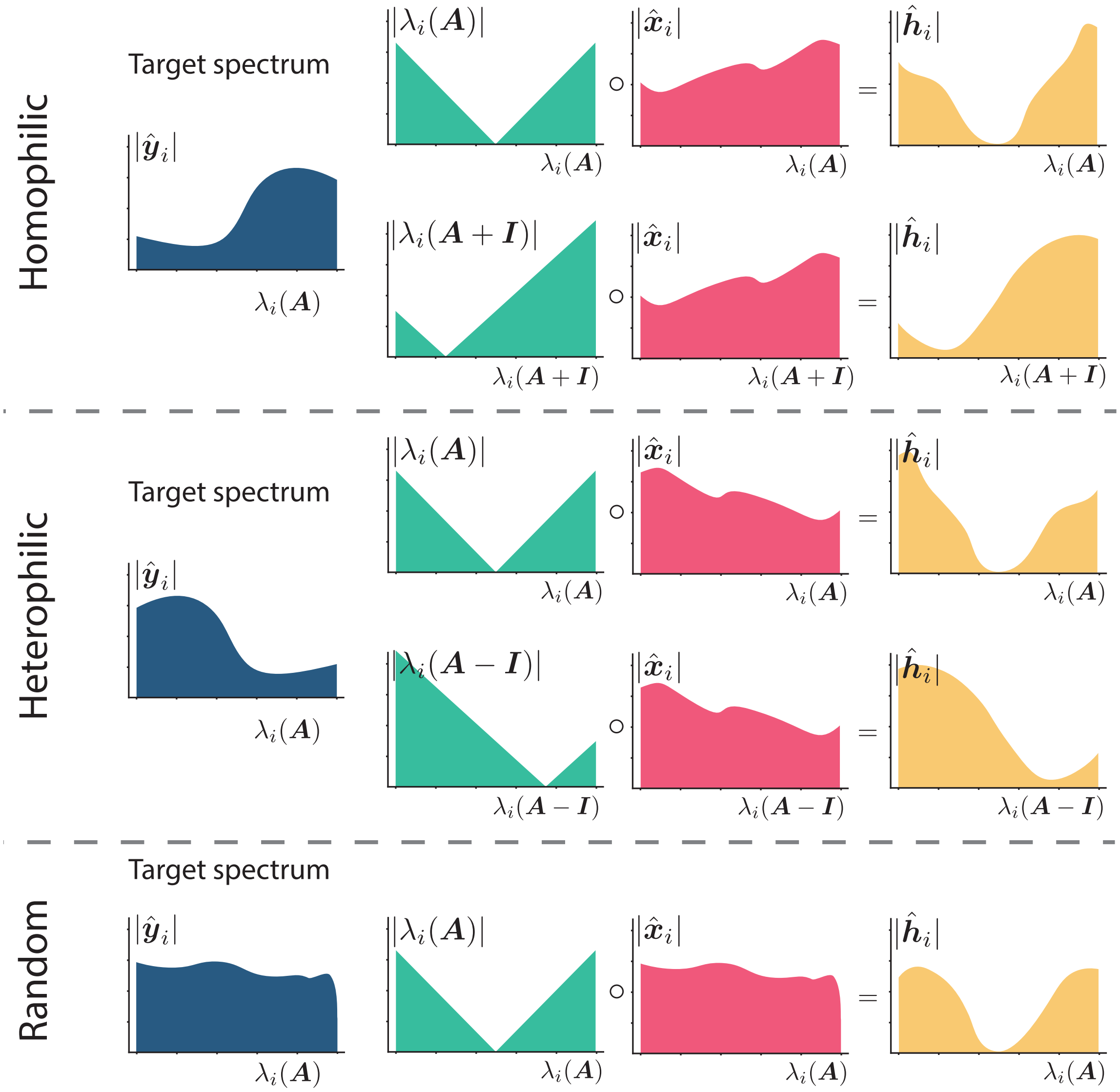

Fig. S2. A spectral perspective understanding of self-loops in GCNs. The first (blue) column shows a projection of the true signal into the graph spectral domain. The following three columns illustrate the process of graph signal filtering $\boldsymbol{A}\boldsymbol{x} = \boldsymbol{h}$ in the graph spectrum domain. The second column shows the eigenvalues for $\boldsymbol{A}$ and $\boldsymbol{A} - \boldsymbol{I}$. The third column shows the signal $\boldsymbol{x}$ in the spectral domain, and the forth column show the corresponding filtered signal in the spectral domain. The signal $\boldsymbol{x} = \boldsymbol{y} + \boldsymbol{\xi}$ is noisy, and it in general becomes closer to the target signal $\boldsymbol{y}$ after been filtered. In the homophilic case, the signal been filtered by $\boldsymbol{A}\boldsymbol{x}$ is closer to the true signal compared to $(\boldsymbol{A} + \boldsymbol{I})\boldsymbol{x}$; while in the heterophilic case, $(\boldsymbol{A} - \boldsymbol{I})\boldsymbol{x}$ is better than $\boldsymbol{A}\boldsymbol{x}$. In all the figures, the spectral basis are arranged in the order of increasing frequency.

References

1. M Mézard, G Parisi, MA Virasoro, *Spin glass theory and beyond: An introduction to the replica method and its applications.* (World Scientific Publishing Company) Vol. 9, (1987).
2. DV Voiculescu, KJ Dykema, A Nica, *Free random variables.* (American Mathematical Soc.) No. 1, (1992).
3. J Pennington, P Worah, Nonlinear random matrix theory for deep learning. *Adv. neural information processing systems* **30** (2017).
4. T Dupic, IP Castillo, Spectral density of products of Wishart dilute random matrices. part i: the dense case. *arXiv preprint arXiv:1401.7802* (2014).
5. DI Shuman, SK Narang, P Frossard, A Ortega, P Vandergheynst, The emerging field of signal processing on graphs: Extending high-dimensional data analysis to networks and other irregular domains. *IEEE signal processing magazine* **30**, 83–98 (2013).
6. A Ortega, P Frossard, J Kovačević, JM Moura, P Vandergheynst, Graph signal processing: Overview, challenges, and applications. *Proc. IEEE* **106**, 808–828 (2018).
7. E Chien, J Peng, P Li, O Milenkovic, Adaptive universal generalized PageRank graph neural network in *International Conference on Learning Representations.* (2021).

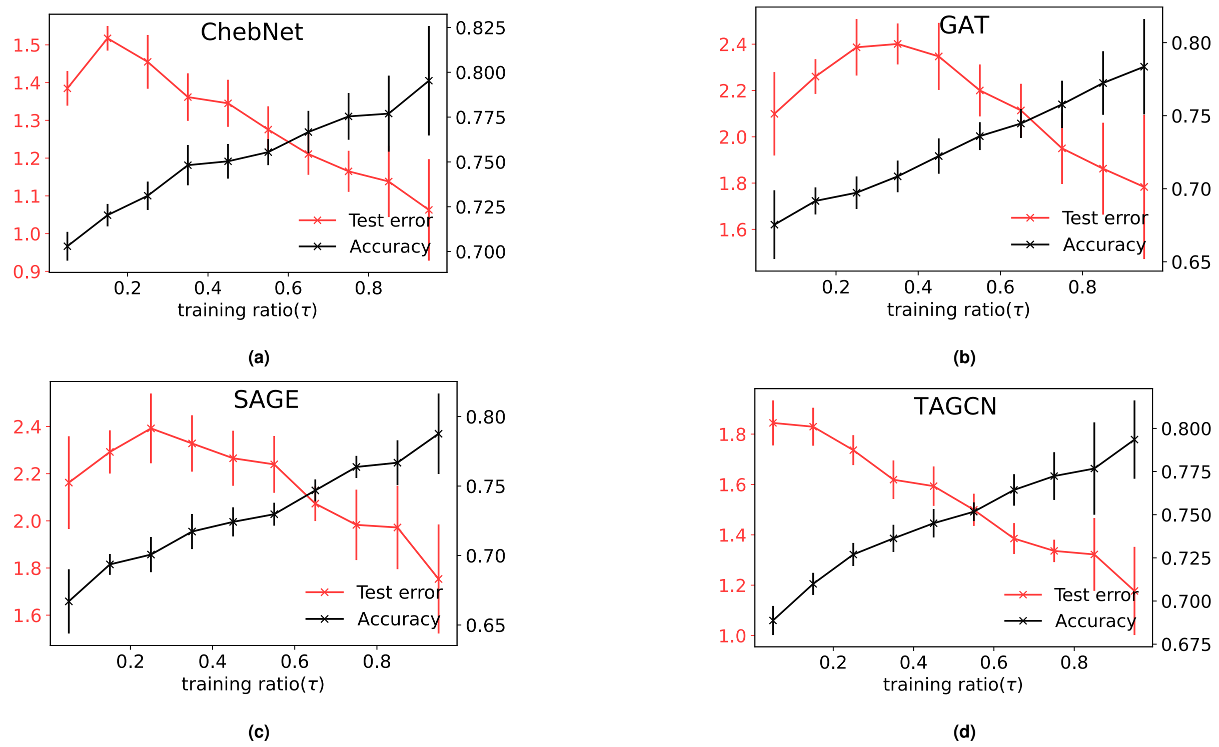

Fig. S3. Test error and classification accuracy at different training ratios for Chebyshev GNN (ChebNet), Graph Attention Network (GAT), the Graph Sample and Aggregate Network (SAGE), Topology Adaptive Graph Convolutional Networks (TAGCN), on the `Citeeer` dataset. All models have two layers with ReLU activations, and are trained by ADAM with the cross-entropy loss.

8. P Veličković, et al., Graph attention networks in *International Conference on Learning Representations.* (2018).
9. W Hamilton, Z Ying, J Leskovec, Inductive representation learning on large graphs. *Adv. neural information processing systems* **30** (2017).
10. M Defferrard, X Bresson, P Vandergheynst, Convolutional neural networks on graphs with fast localized spectral filtering. *Adv. neural information processing systems* **29** (2016).
11. H Pei, B Wei, KCC Chang, Y Lei, B Yang, Geom-GCN: Geometric graph convolutional networks in *International Conference on Learning Representations.* (2020).
